# XIST knockdown suppresses vascular smooth muscle cell proliferation and induces apoptosis by regulating miR-1264/WNT5A/β-catenin signaling in aneurysm

**DOI:** 10.1042/BSR20201810

**Published:** 2021-03-15

**Authors:** Liang Zou, Peng-fei Xia, Lei Chen, Yan-yan Hou

**Affiliations:** 1 Department of Neurosurgery, The Second Clinical Medical School of Inner Mongolia University for Nationalities, Yakeshi, Inner Mongolia, 022150, China; 2 Department of Neurosurgery, Sanbo Brain Hospital Co., Ltd, Beijing, 100093, China; 3 Department of Hematology and Oncology, The Second Clinical Medical School of Inner Mongolia University for Nationalities, Yakeshi, Inner Mongolia, 022150, China

**Keywords:** abdominal aortic aneurysm, lncRNA, miR-1264, proliferation

## Abstract

Long non-coding RNAs (lncRNAs) have been ascertained as vital modulators in abdominal aortic aneurysm (AAA) development. In this research, the function and molecular mechanisms of the lncRNA X-inactive specific transcript (*XIST*) in the evolution of vascular smooth muscle cells (VSMCs) were assessed. Results showed that *XIST* expression was increased but miR-1264 expression level was reduced in the serum of AAA patients. *XIST* depletion impeded human aorta VSMCs (HA-VSMCs’) ability to proliferate and stimulate apoptosis, while repressing miR-1264 expression through an unmediated interaction. Additionally, the influence of *XIST* knockdown on apoptosis and proliferation could be rescued by an miR-1264 inhibitor. Subsequent molecular investigations indicated that *WNT5A* was miR-1264’s target, and *XIST* functioned as a competing endogenous RNA (ceRNA) of miR-1264 to raise *WNT5A* expression. Further, an miR-1264 inhibitor stimulated the proliferation and suppressed the apoptosis of HA-VSMCs through the activation of WNT/β-catenin signaling. Taken together, *XIST* impeded the apoptosis and stimulated the proliferation of HA-VSMCs via the WNT/β-catenin signaling pathway through miR-1264, demonstrating *XIST*’s underlying role in AAA.

## Introduction

Abdominal aortic aneurysm (AAA) is a cardiovascular disease triggering fatal rupture [1,2] and a significant cause of death in the elderly [3]. Infiltration of numerous inflammatory cells [4], raised levels of matrix metalloproteinases (MMPs) [5], excess reactive oxygen species (ROS) [6], medial and intimal calcification [7], new vessel formation [8], vascular smooth muscle cell (VSMC) apoptosis [9], and degeneration of elastic lamellae in the aorta [5] have been shown to participate in aneurysms. Although the disease manifests no symptoms prior to rupture, rupture in AAA cases often leads to death, with a mortality rate of 85–90% [10]

SMC apoptosis is an important pathological feature that leads to various mechanisms that modulate AAA [9]. Recently, emphasis has been placed on the underlying mechanisms of SMC apoptosis for its contribution to the diagnosis and treatment of AAA. Many recent studies have indicated that various proteins can be used as biomarkers and therapeutic targets in AAA diagnosis and treatment due to their regulation of SMC apoptosis [11–13].

Long non-coding RNAs (lncRNAs) are RNAs that are longer than 200 nucleotides, are identical with mRNAs in their processing and transcription, but are unable to encode proteins [14,15]. Mounting recent evidence has suggested that a considerable number of lncRNAs are pivotal modulators that participate in specific physiological and pathological processes through transcriptional or post‐transcriptional modulating mechanisms [16]. Numerous studies have shown that dysregulation of lncRNAs are associated with different diseases including cancers [17], neurodegenerative diseases [18], cardiovascular diseases, inflammatory diseases and pulmonary fibrosis. Additionally, multiple lncRNAs have been shown to facilitate SMC apoptosis in AAA formation [19,20], and the lncRNA H19 was recently implicated in AAA advancement through its modulation on SMC survival [21]. Nonetheless, the roles of other lncRNAs in AAA pathogenesis as well as their therapeutic potential remain elusive. The lncRNA X-inactive specific transcript (*XIST*), which is located on the X chromosome [22,23], is abundantly present in multiple cancers and modulates tumor formation and growth as a potential oncogene through competing RNA mechanisms [24,25]. *XIST* has been ascertained to be involved in cellular biological processes such as genome maintenance, differentiation and proliferation [26]. Here, it was hypothesized that *XIST* may modify proliferation and induce the apoptosis response in AAA.

MiRNA, a type of ncRNA with 18–25 nucleotides, contributes to mRNA degradation by directly interacting with their 3′ untranslated regions (UTRs) [27]. The competing endogenous RNA (ceRNA) hypothesis speculates that lncRNAs serve as miRNA sponges, thus participating in the progression of cancers by modulating the expression of miRNA’s target genes [28]. However, whether *XIST* can interact with miRNAs to regulate the development of AAA remains to be elucidated. In the present study, we confirmed *XIST* could sponge miR-1264. Previous studies revealed that down-regulation of miR-1264 contributes to DNMT1-mediated silencing of SOCS3 and affect the smooth muscle cell proliferation [29]. In colorectal cancer, lncRNA SOCS2-AS1 inhibits tumor progression and metastasis via sponging miR-1264 [30]. However, the role of miR-1264 played in AAA remained unclear.

Thus, our research aims to study the possible functions and molecular bases of *XIST* and miR-1264 in VSMC progression, so as to find underlying targets for AAA treatment.

## Materials and methods

### Clinical specimens

Twenty-four AAA patients not undergoing treatment and twenty-four healthy volunteers were enrolled in the present study. Participants were aged between 50 and 70 and 30% were female. This research gained the approval of The Second Clinical Medical School of Inner Mongolia University for Nationalities, and all research subjects signed a written informed consent form. Inclusion standards for the healthy volunteers were as follows: they did not suffer from AAA disease, inflammatory disease, malignant tumors, recent infection (<1 month), or autoimmune diseases. Blood samples (10 ml) were collected from all participants and placed in centrifuge tubes with no anticoagulant. The collected blood samples were maintained at room temperature for approximately 1 h and centrifuged at 3000 rpm for 5 min to extract serum. Finally, TRIzol reagent, provided by Invitrogen (U.S.A.), was applied to separate RNAs in the serum.

### Cell culture

Human aorta VSMCs (HA-VSMCs) were bought from ATCC (U.S.A.) and grown in F-12 K medium (ATCC) containing 0.05 mg/ml ascorbic acid (Sigma–Aldrich (U.S.A.)), 10% fetal bovine serum (Invitrogen), 0.01 mg/ml insulin (Sigma–Aldrich), 0.01 mg/ml transferrin (Sigma–Aldrich), 10 ng/ml sodium selenite (Sigma–Aldrich), 10 mM HEPES (Sigma–Aldrich), 10 mM TES (Sigma–Aldrich) and 0.03 mg/ml endothelial cell growth supplement (Cell Application (U.S.A.)) in a humid incubator with 5% CO_2_ at 37°C.

### Cell transfection and treatment

Polymerase chain reaction (PCR) amplification was executed for full-length *XIST* sequences, and these sequences were subcloned into pcDNA3.1 vectors acquired from Invitrogen to construct pcDNA-*XIST* overexpression plasmids. GenePharma Co. Ltd (China) designed and synthesized small interfering RNA (siRNA) targeting *XIST* (si-XIST#1 and si-XIST#2) and its negative control (si-NC), siRNA against WNT family member 1 (si-*WNT5A*) and its scramble control (scramble NC), miR-1264 inhibitor and its negative control (inhibitor NC), and miR-1264 mimic (miR-1264) and its negative control (mimic NC). Cells were transfected with Lipofectamine 2000 (Invitrogen) as per the guidance of manufacturer. XAV939, an inhibitor of β-catenin, was commercially provided by Sigma–Aldrich.

### Quantitative reverse transcription-PCR

Total RNA was extracted from serum using TRIzol® reagent (Invitrogen) according to manufacturer’s instructions. Equal amounts of RNA from each sample (1 μg) underwent reverse transcription (RT) into first-strand cDNA using an M-MLV reverse transcriptase acquired from Invitrogen and specific RT primers (miR-1264 and U6 snRNA) or random primers (*XIST* and GAPDH). Then, the expression of *XIST*, GAPDH, miR-1264 and U6 snRNA were examined with the use of the SYBR Green Real-Time PCR Master Mix from TOYOBO (Japan) and qPCR primers. Ribobio Co., Ltd. (China) synthesized miR-1264 and U6 snRNA primers (RT primers and qPCR primers). Roche Lightcycler 480 (U.S.A.) was applied for quantitative reverse transcription-PCR (qRT-PCR). *GAPDH* was used as an endogenous control gene for *XIST*, while U6 was an endogenous control for miR-1264.

The qPCR primers for *XIST* and GAPDH are as follows:

*XIST*: 5′-GACACAAGGCCAACGACCTA-3′ (F),

5′-TCGCTTGGGTCCTCTATCCA-3′ (R);

GAPDH: 5′-CCTGGCCAAGGTCATCCATG-3′ (F),

5′-GGAAGGCCATGCCAGTGAGC-3′ (R).

### Western blotting

RIPA lysis buffer (Beyotime) was utilized to extract whole proteins from the serum, which were quantified with a Pierce BCA Protein Assay Kit from Thermo Fisher Scientific (U.S.A.). Then, 50 μg of protein from each sample was isolated through SDS/PAGE and transferred on to PVDF membranes from Millipore (U.S.A.). Membranes were incubated in 5% fat-free milk for 1 h at room temperature, followed by overnight incubation at 4°C with primary antibodies against GAPDH, proliferating cell nuclear antigen (PCNA), Bax, Bcl-2, Ki-67, β-catenin, *WNT5A*, E-cadherin and C-myc. Next, the membranes were further incubated for 1 h at room temperature with a secondary antibody conjugated with horseradish peroxidase. Clarity Max™ Western ECL Substrate (Bio-Rad, U.S.A.) was employed to visualize protein signals. Abcam (U.K.) commercially provided all the above antibodies.

### CCK-8 assay

Transfected HA-VSMCs were plated in a 12-well plate and grown for 15 days in a complete medium. Cells were fixed with methanol and dyed with 0.1% Crystal Violet solution (Sigma–Aldrich). A microscope was utilized to count colonies with at least 50 cells.

### Annexin V assay

Cells (∼1 × 10^6^) were harvested and washed. The washed cells were resuspended in PBS containing 40 μg/ml propidium iodine and 100 g/ml RNaseA (Sigma–Aldrich; Merck KGaA) without calcium and magnesium. Cells were incubated at 37°C for 30 min avoiding light. A nylon mesh sieve was applied to remove cell clumps from stained cells, and then an FACScan flow cytometer and the CELL QUEST analysis software (Becton Dickinson, Inc.) were used to analyze cell apoptosis.

### Luciferase activity assay

PCR amplification was executed for fragments of *XIST* and *WNT5A* 3′ UTRs with miR-1264 binding sites. The fragments were established in the psiCHECK-2 vector (Promega, U.S.A.) to generate *XIST*-WT and *WNT5A*-WT reporters. The reporters with miR-1264 binding sites were also produced using Quickchange Multi Site-Directed Mutagenesis kit acquired from Stratagene (U.S.A.). HA-VSMCs were treated with the established luciferase reporters with plasmids or miRNAs, independently. Forty-eight hours later, a dual luciferase reporter assay kit (Promega) was utilized for the examination of luciferase activity in cells following the manufacturer’s instructions.

### AAA mouse model

Animal experiments were performed at the SPF Animal Laboratory at Inner Mongolia University for Nationalities. All animal experiments were approved by the Institutional Animal Care and Use Committee of Inner Mongolia University for Nationalities (No. WZ2018026) and were carried out in accordance with the guidelines for the Care and Use of Laboratory Animals of the National Institutes of Health. All the Apolipoprotein E deficient (ApoE^−/−^) mice on a C57BL/6J genetic background (male, 12 weeks old) were obtained from GemPharmatech (Nanjing, China), and were raised in SPF-grade environment with a 12-h light/dark cycle. ApoE^−/−^ mice were infused with Ang-II (Sigma–Aldrich, MO, U.S.A.) at a rate of 1 μg/kg/min during 28 days to establish AAA mouse model (the aortic diameter has increased 50% from the initial diameter). Equal volume of 0.9% NaCl was induced in the normal group. Then AAA mouse model was transfected with the lentiviral shRNA-XIST and the corresponding negative control, which were synthesized by Genepharma (Shanghai, China). The mice were anesthetized by intraperitoneal injection of sodium pentobarbital (40 mg/kg) before operation to relieve the pain. All animals were killed by an overdose (>120 mg/kg body weight) via intraperitoneal injection of pentobarbital. Mortality was verified by loss of spontaneous breathing.

### Histological analysis

Mouse aortic tissues were isolated and fixed with 4% paraformaldehyde at 4°C for 12–24 h. Aortic tissues were then dehydrated and embedded in paraffin. Six-micrometer-thick paraffin sections were used to conduct Hematoxylin–Eosin (HE) staining according to the manufacturer’s instructions and the HE staining kit was purchased from BOSTER Biological Technology (Wuhan, China).

### Statistical analysis

Data presented reflect the mean ± SEM from independent experiments that were carried out at least thrice. Differences among various groups were examined via a one-way variance analysis or Student’s *t* test. Statistical significance was regarded as *P*<0.05.

## Results

### XIST expression is elevated while miR-1264 expression is reduced in the serum of AAA patients due to a direct interaction

**Figure 1:**
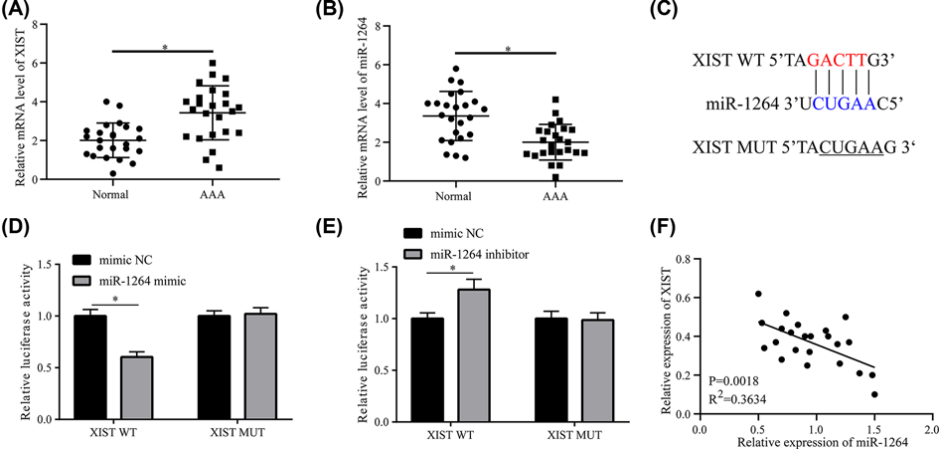
*XIST* expression is elevated while miR-1264 expression is reduced in the serum of AAA patients due to a direct interaction (**A**) Expression of *XIST* and (**B**) miR-1264 in serum from AAA patients (*n*=24) and healthy participants (*n*=24). (**C**) The predicted binding sites between miR-1264 and *XIST*, and the mutant sites in the *XIST*-MUT reporter are shown. (**D,E**) Luciferase activity in HA-VSMCs co-treated with *XIST*-WT or *XIST*-MUT reporters and miR-1264 or its scramble control (mimic NC or inhibitor NC). (**F**) A negative correlation between *XIST* and miR-1264 expression in the serum of 24 AAA patients. Data are presented as mean ± SEM (*n*=3) of at least three independent assays. *P<0.05.

*XIST* and miR-1264 expression levels were first explored in the serum of AAA patients. qRT-PCR assay findings uncovered that *XIST* expression was increased (Figure 1A) while miR-1264 expression was pronouncedly reduced (Figure 1B) in the serum of AAA patients (*n*=24) relative to that of healthy volunteers (*n*=24). Bioinformatics analysis (https://portlandpress.com/pages/figure_guidelines) was utilized to confirm the underlying miRNA targets for *XIST* to probe at the molecular mechanisms by which *XIST* regulates the development of HA-VSMCs. Complementary sites between miR-1264 and *XIST* were identified (Figure 1C). These findings imply that *XIST* and miR-1264 might be pivotal interactors in AAA development. With the aim of continuously verifying this speculation, HA-VSMC cells were treated with a constructed *XIST*-WT or *XIST*-MUT luciferase reporter with miR-1264 or its scramble control (mimic NC or inhibitor NC). The luciferase reporter assay showed that the presence of miR-1264 resulted in dramatic loss of reporter activity in *XIST*-WT cells relative to the scramble control. Nevertheless, miR-1264 did not affect the reporter activity of the *XIST*-MUT reporter, due to the mutation being located within the predicted binding sites between miR-1264 and *XIST* (Figure 1D). Moreover, the introduction of an miR-1264 inhibitor caused an increase in luciferase activity in the *XIST*-WT reporter relative to the scramble NC (Figure 1E). Furthermore, it was uncovered that miR-1264 expression had an inverse relationship to *XIST* level in the serum of 24 AAA patients (Figure 1F). Thus, *XIST* impedes miR-1264 expression through a mutual interaction.

### Down-regulation of *XIST* inhibited the morbidity and development of AAA

**Figure 2:**
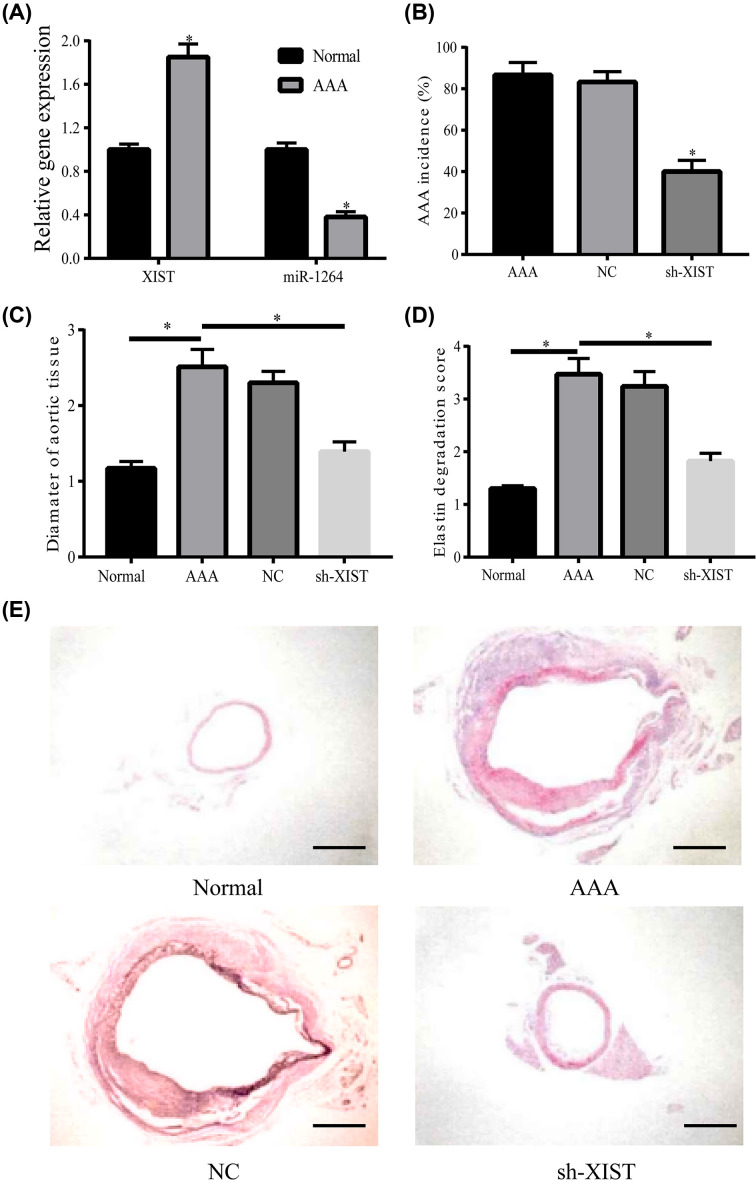
Down-regulation of *XIST* inhibited the morbidity and development of AAA (**A**) *XIST* was up-regulated in AAA mice model, but miR-1264 was down-regulated. (**B**) The incidence rate of AAA in each group was analyzed. (**C**) The maximum diameter of each aortic tissue was measured. (**D**) The elastin filament degradation score in each group (*n*=10 aorta/group). (**E**) Hematoxylin staining was performed to detect the pathological change in aortic tissues in each group. *P<0.05.

Subsequently, we constructed AAA mice model and found that *XIST* was up-regulated and miR-1264 was down-regulated in AAA mice model tissues compared with the normal mice (Figure 2A). In order to investigate the role of *XIST* in AAA formation, sh-*XIST* and NC were injected into AAA model mice (*n*=20 per group). Finally, aortic tissues were collected from each mouse. Low-expression of *XIST* reduced the incidence of AAA and the aortic maximum diameter of AAA mice (Figure 2B,C). Arterial wall elastic fibers were also damaged more seriously in sh-*XIST* group than the normal and NC mice (Figure 2D). After *XIST* was knocked down, the lesions in the aortic tissues from AAA mice model were also attenuated obviously (Figure 2E).

### XIST knockdown prevents HA-VSMC proliferation and triggers apoptosis

**Figure 3:**
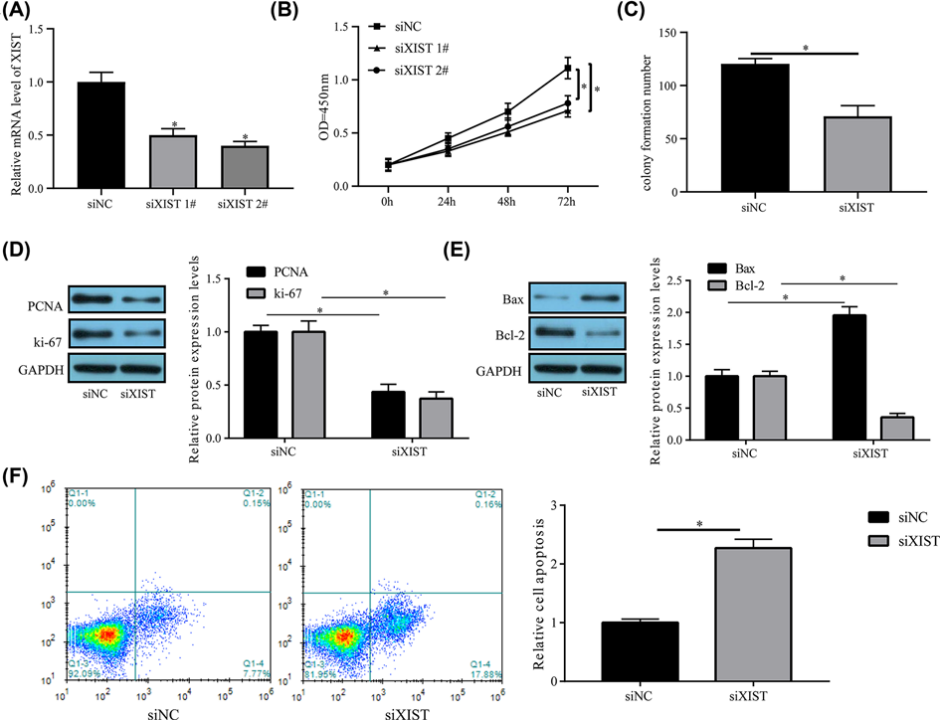
*XIST* knockdown represses HA-VSMCs proliferation and triggers apoptosis HA-VSMCs transfected with si-NC, si-*XIST*#1 or si-*XIST*#2 were assessed for (**A**) *XIST* expression (**B**) proliferative capacity, (**C**) clone formation ability, (**D**) PCNA and Ki-67 expression, and (**E**) Bcl-2 and Bax expression and the relative cell apoptosis (**F**). Data are reflected as mean ± SEM (*n*=3) of at least three independent assays. *P<0.05.

The role of *XIST* in the proliferation and apoptosis of HA-VSMCs was assessed by obstructing *XIST* expression using siRNAs (siXIST #1 and siXIST#2) to probe into *XIST*’s role in AAA advancement. Cell proliferation and apoptosis were subsequently examined. According to qRT-PCR findings, siXIST#1 and siXIST#2 caused the knockdown of *XIST* expression relative to si-NC in HA-VSMCs (Figure 3A). A CCK-8 assay uncovered that *XIST* knockdown overtly repressed cell proliferation relative to the scramble NC (Figure 3B). Further, *XIST* knockdown also overtly weakened the capacity of HA-VSMCs to form colony relative to the scramble NC (Figure 3C). In line with expectations, PCNA and Ki-67 expression were evidently reduced in HA-VSMCs after *XIST* knockdown (Figure 3D). Additionally, Bax expression was increased while Bcl-2 expression was strikingly reduced in HA-VSMCs treated with *XIST* siRNAs (Figure 3E). Annexin V assay showed the relative apoptotic cells in siXIST group was more than the siNC group (Figure 3F). In summary, *XIST* reduction impeded HA-VSMCs proliferation ability and stimulated apoptosis.

### MiR-1264 inhibitor reduced the effect of XIST knockdown on the proliferation and apoptosis of HA-VSMCs

**Figure 4:**
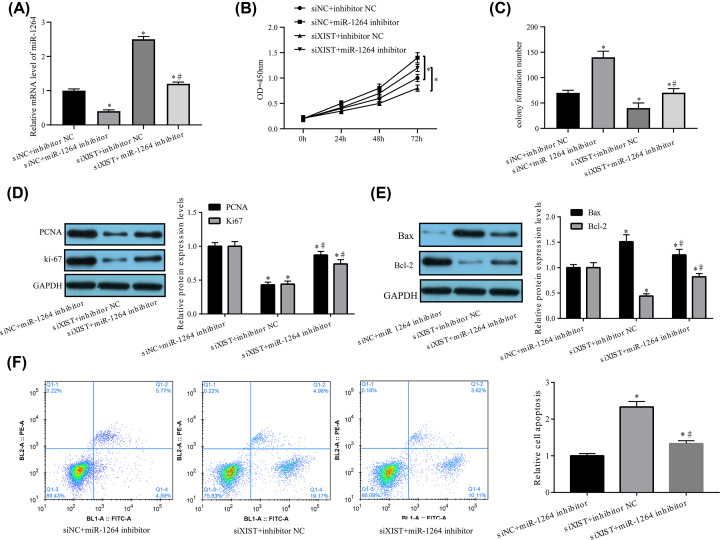
miR-1264 inhibitor reverses the effect of *XIST* knockdown on HA-VSMC apoptosis and proliferation HA-VSMCs treated with si-NC + inhibitor NC, si-NC + miR-1264 inhibitor, si-*XIST* + inhibitor NC, or si-*XIST* and miR-1264 inhibitor were assessed for (**A**) miR-1264 expression, (**B**) cell proliferation ability and (**C**) colony formation capacity. HA-VSMCs treated with si-NC + miR-1264 inhibitor, si-*XIST* + inhibitor NC or si-*XIST* and miR-1264 inhibitor, were assessed for (**D**) expression of Ki-67, PCNA, (**E**) Bax and Bcl-2 and the relative cell apoptosis (**F**). Data are reflected as mean ± SEM (*n*=3) of at least three independent assays. *,^#^*P*<0.05 (*, compared with si-NC + inhibitor NC or si-NC + miR-1264 inhibitor; ^#^, compared with si-*XIST* + inhibitor NC).

To corroborate whether miR-1264 mediated the impact of *XIST* knockdown, miR-1264 expression in HA-VSMCs treated with si-*XIST* was inhibited. A qRT-PCR assay uncovered that an miR-1264 inhibitor led to decreased miR-1264 expression and abated the promotion of si-*XIST* on miR-1264 expression in HA-VSMCs (Figure 4A). Thereafter, CCK-8, Western blotting and colony formation experiments further disclosed that the miR-1264 inhibitor prominently abated the repression of *XIST’s* diminution on cell growth, manifested as increased cell proliferation (Figure 4B) and colony formation (Figure 4C) as well as elevated PCNA and Ki-67 expression (Figure 4D). MiR-1264 depletion also reduced Bax expression but raised Bcl-2 expression (Figure 4E) in HA-VSMCs treated with si-*XIST* which was consistent with the Annexin V assay (Figure 4F). Altogether, the above findings denote that miR-1264 modulated the proliferation inhibition and apoptosis promotion of HA-VSMCs resulting from *XIST* knockdown.

### WNT5A is a target of miR-1264

**Figure 5:**
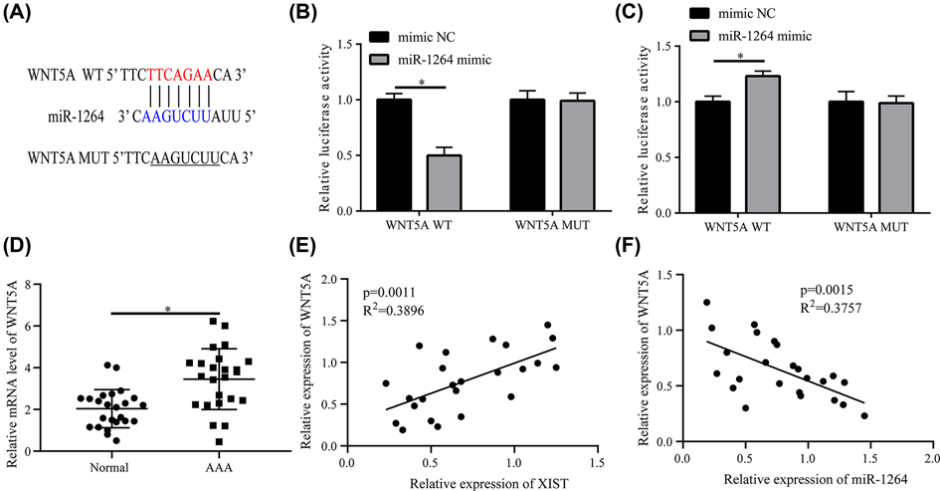
*WNT5A* is a miR-1264 target (**A**) Predicted binding sequences between miR-1264 and *WNT5A* 3′ UTR region with mutant sites in the *WNT5A*-MUT reporter. (**B,C**) Luciferase activity test following co-treatment of HA-VSMCs with *WNT5*-WT or *WNT5*-MUT reporter and miR-1264 or miR-1264 inhibitor for 48 h. (**D**) *WNT5A* expression in the serum of healthy participants (*n*=24) and AAA patients (*n*=24). (**E,F**) Relationship between *WNT5A* and *XIST* or miR-1264 in the serum of 24 AAA patients is analyzed. Data are reflected as mean ± SEM (*n*=3) of at least three independent assays. *P<0.05.

Considerable research demonstrates that lncRNAs modulate target mRNA expression by functioning as miRNA ceRNAs [31]. Therefore, the possible targets of miR-1264 were inquired using TargetScan software. Figure 5A shows the probable binding sequences of miR-1264 in the 3′ UTR region of *WNT5A*. A luciferase experiment corroborated that enforced miR-1264 expression weakened the luciferase activity of the *WNT5A*-WT reporter. Nevertheless, the luciferase activity of the *WNT5A*-MUT reporter did not change following miR-1264 overexpression; however, the activity of the *WNT5A*-WT reporter in an miR-1264 inhibitor was enhanced (Figure 5B,C). It was also discovered that *WNT5A* was highly expressed in the serum of 24 AAA patients compared with the healthy participants (Figure 5D). Further, qRT-PCR experiments showed that *WNT5A* expression was positively associated with *XIST* expression (Figure 5E) but had an inverse association with miR-1264 expression (Figure 5F) in the serum of AAA patients. Thus, it could be inferred from these findings that *XIST* elevates *WNT5A* expression in HA-VSMCs by functioning as an miR-1264’s ceRNA.

### MiR-1264 curbs HA-VSMC proliferation and promotes apoptosis via WNT/β-catenin signaling

**Figure 6:**
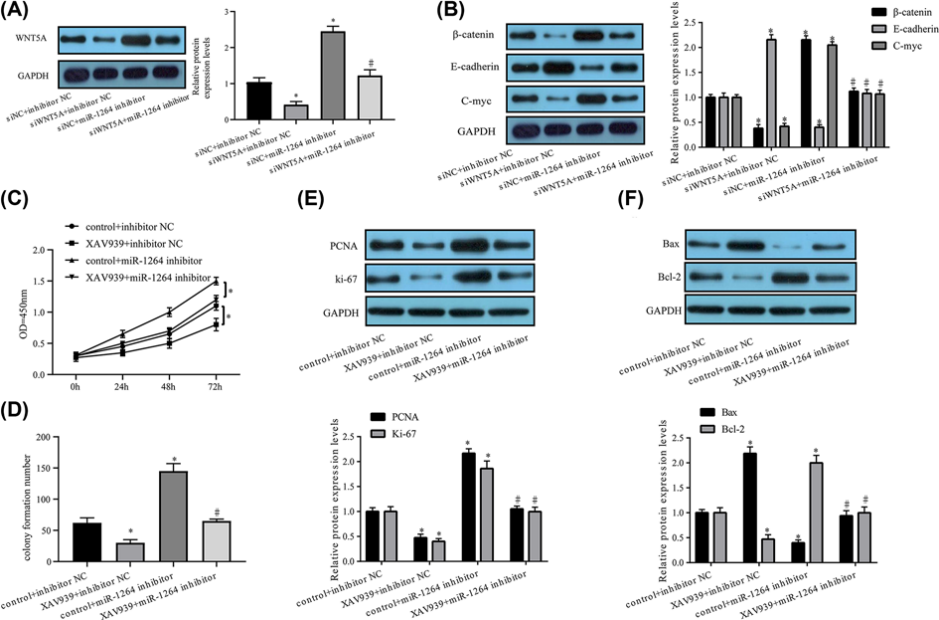
miR-1264 modulates HA-VSMC proliferation and apoptosis through WNT/β-catenin signaling (**A,B**) HA-VSMCs transfected with si-NC + inhibitor NC, si*WNT5A* + inhibitor NC, si-NC + miR-1264 inhibitor or si-*WNT5A* + miR-1264 inhibitor were transfected for 24 h and the expression of *WNT5A*, β-catenin, E-cadherin and C-myc were assessed. HA-VSMCs underwent transfection with NC inhibitor or miR-1264 inhibitor with or without XAV939 (10 μM) for 10 h, followed by tests for (**C**) cell proliferation capacity, (**D**) colony formation capacity and the expression of (**E**) Ki-67, PCNA, and (**F**) Bcl-2 and Bax. Data are reflected as mean ± SEM (*n*=3) of at least three independent assays. *,^#^*P*<0.05 (*, compared with si-NC + inhibitor NC or control+ inhibitor NC; ^#^, compared with si*WNT5A* + inhibitor NC or XAV939+ inhibitor NC).

*WNT5A* belongs to the WNT family; WNT signaling is a crucial regulating pathway in AAA advancement. Hence, the influence of *WNT5A* and miR-1264 on the WNT signaling pathway in HA-VSMCs was assessed. Western blotting uncovered that si-*WNT5A* triggered a notable decline in *WNT5A* expression, and *WNT5A* knockdown overtly abated the elevated *WNT5A* expression in HA-VSMCs mediated by an miR-1264 inhibitor (Figure 6A). Then, the effect of si-*WNT5A* on the expression of genes (*β-catenin, C-myc, E-cadherin*) associated with WNT signaling in HA-VSMCs was assessed. The miR-1264 inhibitor caused an increase in β-catenin and C-myc expression and a decrease in E-cadherin, all of which were evidently abrogated after *WNT5A* knockdown (Figure 6B), revealing that miR-1264 regulates WNT/β-catenin signaling. Thereafter, the role of miR-1264 in WNT/β-catenin signaling was examined in HA-VSMCs. HA-VSMCs were treated with miR-con or miR-1264 with or without the β-catenin inhibitor XAV939 (10 μM) for 10 h. According to the findings, XAV939 treatment overtly impeded cell proliferation (Figure 6C,D) as well as PCNA and Ki-67 expression (Figure 6E). In contrast, miR-1264 reduction increased cell proliferation, which was prominently abated after XAV939 treatment (Figure 6C–E). Further, XAV939 facilitated HA-VSMC apoptosis and increased Bax expression while reducing Bcl-2 expression. However, miR-1264 reduction decreased HA-VSMC apoptosis, which was impeded by XAV939 treatment (Figure 6). At length, miR-1264 modulated WNT/β-catenin signaling to impede HA-VSMC proliferation and stimulate apoptosis.

## Discussion

AAA is a common life-threatening disease that affects 1–2% of men by the age of 65 [32]. The most serious complication of AAA is rupture, which is usually fatal, with a reported age-adjusted annual mortality of 15.1 per 1 million people in the United States [33].

As an *XIST* gene product, lncRNA*-XIST* is a major modulator of X-inactivation in mammals, and the *XIST* gene is exclusively transcribed from the inactive X chromosome [22]. *XIST* is highly expressed in cases of glioblastoma [24], breast cancer [34] and ovarian cancer [35], suggesting it may be a biomarker for cancer diagnosis [36]. It has recently been evidenced that *XIST* is crucial for long-term survival of hematopoietic stem cells [37]. Further, *XIST* reduction represses tumors through decreasing cell proliferation, invasion and migration, and triggering apoptosis. *In vivo* research also uncovered that *XIST* knockdown impeded tumor growth and prolonged the survival of nude mice [24]. Thus, *XIST* influences the onset and advancement of malignancies, but the mutual action between *XIST* and AAA advancement has not yet been reported. In the present study, we first found that *XIST* was up-regulated in AAA patients’ serum and AAA mice model artery tissues.

The AAA main pathological changes include chronic inflammation, blood vessel walls elastic fibrous fracture degradation and middle artery outer membrane of freshman blood vessel formation. The dysregulated proliferation and apoptosis of smooth muscle cells might lead to the blood vessel formation and several lines of evidence have shown the proliferation and apoptosis of smooth muscle cells in AAA tissues is abnormal. Therefore, it is necessary to explore the effects of *XIST* played on the proliferation and apoptosis of smooth muscle cells. As results have shown, the knockdown of *XIST* could inhibit HA-VSMCs proliferation but promote apoptosis. *In vivo*, we found that down-regulated *XIST* reduced the incidence of AAA and the aortic maximum diameter of AAA mice, indicating *XIST* might play a vital role in AAA.

To explore the underlying mechanism of *XIST* in AAA, bioinformatics analysis was employed and miR-1264 was the potential target gene of *XIST.* Luciferase reporter assays and qRT-PCR experiments ascertained that *XIST* was capable of interfering with miR-1264 expression through a direct interaction. MiRNAs exerted pivotal effects on SMC fate and behavior [38]. It has been ascertained that miRNAs participate in various cellular functions, including differentiation, growth and development in vascular diseases [1,39]. In this study, miR-1264 expression was negatively associated with *XIST* expression in the serum of AAA patients and was also down-regulated in AAA mice model artery tissues The deficiency of miR-1264 reversed the effects of proliferation inhibition and apoptosis promotion by *XIST* knockdown in HA-VSMCs indicating miR-1264 mediated *XIST*’s influence on the procedure of AAA.

There are mounting evidences that miRNAs function by modulating the stability or translation of target mRNAs. Therefore, possible targets of miR-1264 were sought using TargetScan software, the findings of which revealed *WNT5A* as a possible target of miR-1264 target. This was further confirmed by the following luciferase assay. It has been previously reported that WISP-1 induced by *WNT5A* curbs VSMC apoptosis triggered by oxidative stress [40], *WNT5A* is induced by TGF-β/Smad3 in rat aortic SMCs and promotes their proliferation [41]. It is also associated with the differentiation of bone marrow mesenchymal stem cells in vascular calcification by linking various receptors [42]. The current research showed that *XIST* elevated *WNT5A* expression in HA-VSMCs by functioning as a ceRNA of miR-1264. Also, *WNT5A* mRNA expression was positively associated with *XIST* expression and had an inverse correlation with miR-1264 expression in the serum of AAA patients.

Previous research has shown that *WNT5A* triggers the activation of β-catenin [43] and C-myc signaling in VMSCs, facilitates proliferation while limiting apoptosis in rat VSMCs [44], and activates WNT/β-catenin signaling which decreases E-cadherin expression [45]. WNT/β-catenin signaling pathway plays an important regulatory role in physiological processes such as cell differentiation, proliferation and apoptosis. The essential functions of the WNT/β-catenin signaling pathway and cadherin–catenin complex in VSMC development have been summarized in a previous review [46]. Considering the important role of WNT/β-catenin signaling pathway played in VSMC development and cell proliferation and apoptosis, the WNT/β-catenin pathway might be involved in the procedure of AAA. The influence of *WNT5A* and miR-1264 on the expression of genes (*β-catenin, E-cadherin and C-myc*) involved in WNT signaling in HA-VSMCs were examined further. According to the findings, *WNT5A* reduction impeded WNT/β-catenin signaling, while miR-1264 deficiency led to WNT/β-catenin pathway activation in HA-VSMCs. Besides, *WNT5A* repression undermined the actions of the miR-1264 inhibitor on WNT/β-catenin signaling. The above findings elaborated that miR-1264 inhibition modulates *WNT5A* to activate WNT/β-catenin signaling. Subsequently, whether miR-1264 influenced the apoptosis and proliferation of HA-VSMCs via the WNT/β-catenin pathway was analyzed. Results denoted that miR-1264 depletion increased proliferation ability and blocked apoptosis of HA-VSMCs, and these influences were pronouncedly dampened by XAV939 treatment. At length, miR-1264 repression regulates WNT/β-catenin signaling to facilitate HA-VSMCs to grow and limit apoptosis.

## Conclusion

This research corroborated that *XIST* displayed a specific expression in the abdominal aorta, and decreased *XIST* expression stimulated SMC apoptosis but impeded proliferation, which then mediated AAA formation. In terms of the mechanism for this process, *XIST* likely plays a part in AAA formation by inactivating WNT/β-catenin signaling and sponging miR-1264, implying the potential use of *XIST* in AAA prevention.

## Data Availability

The datasets used and/or analyzed during the present study are available from the corresponding author on reasonable request.

## Abbreviations

AAA: abdominal aortic aneurysm
ApoE^−/−^: Apolipoprotein E deficient
*CCK-8*: Cell Counting Kit-8
ceRNA: competing endogenous RNA
*DNMT1*: DNA methyltransferase 1
HA-VSMC: human aorta VSMC
HE: Hematoxylin–Eosin
lncRNA: long non-coding RNA
PCNA: proliferating cell nuclear antigen
PCR: polymerase chain reaction
qRT-PCR: quantitative reverse transcription-PCR
siRNA: small interfering RNA
UTR: untranslated region
VSMC: vascular smooth muscle cell
*XIST*: X-inactive specific transcript
